# Description of a new species *Gyraulus* (Pulmonata: Planorbidae) from the land thermal spring Khakusy of Lake Baikal

**DOI:** 10.3897/zookeys.762.23661

**Published:** 2018-05-30

**Authors:** Tatiana Sitnikova, Tatiana Peretolchina

**Affiliations:** 1 Limnological Institute SB RAS, Ulan-Batorskaya Str., 3, Irkutsk, 664033, Russia

**Keywords:** COI mtDNA, morphology, planorbid gastropods

## Abstract

A new species of the family Planorbidae is described from the land thermal spring Khakusy, on the north-eastern shore of Lake Baikal. The description of *Gyraulus takhteevi* **sp. n.** includes morphological characters and gene sequences (COI of mtDNA) for the species separation from sister taxon *Gyraulus acronicus* (A. Férussac, 1807) collected from the small Krestovka River in-flowing into the south-western part of the Lake. The new species differs from *G. acronicus* in small shell size of adults, having smaller number of prostate folds (maximal up to 26 in *G. takhteevi* n. sp. vs. 40 in *G. acronicus*), a short preputium (approximately twice shorter than the phallotheca), and an elongated bursa copulatrix. The population of *Gyraulus takhteevi* **sp. n.** consists of two co-existent morphs: one of them has a narrow shell spire and the second is characterized by wide spire similar to the shell of *G. acronicus*. One of the two revealed haplotypes of the new species includes both morphs, while the second consists of snails with wide spired shells.

## Introduction

The thermal spring Khakusy is located one kilometer from the shore in the north-eastern part of Lake Baikal and flows into it. The chemical composition of the water is hydrocarbonate-sulphate-sodium with a salinity of 0.3 g/l; the water temperature of the mainstream is + 47 °C (Volkov 2010). Bottom ground of this warm stream is sand and pebbles, covered with mats of blue-green algae. Gastropods (limnaeids and planorbids) live far from the main stream, where the water temperature varies from + 31 °C to 10 °C. Among limnaeids two species, *Radix khakusyensis* Kruglov & Starobogatov, 1989 and *Radix thermobaicalica* Kruglov & Starobogatov, 1989, were described, which are now proposed to be considered as ecotypes of *Radix auricularia* (Linnaeus, 1758) (Aksenova et al. 2017). Together with *Radix*, small specimens of *Gyraulus* (Planorbidae) were found, which differed from all other species of *Gyraulus* inhabiting the Baikal region including the thermal springs (Sitnikova and Takhteev 2006). Unique morphological characters of these snails support to their status as a new species.

## Materials and methods

Two hundred fourteen specimens of a new species were collected in the thermal spring Khakusy (north-eastern shore of Lake Baikal (55°21'42"N, 109°49'41"E), from pebbles covered by vegetation, mainly filamentous cyanobacterial mats. The samples were collected on 30 March 1990 (13 specimens) from spring with a water temperature of +31 °C, and 39 specimens from slightly downstream with water temperature at +22 °C by V. Takhteev; 3 July 2003 (6 spec.) by T. Sitnikova; 7 October 2004 (23 specimens) by V. Takhteev; 9 August 2009 (14 specimens, 6 dissected) by T. Sitnikova, 20 March 2003 (61 specimens) by V. Takhteev; 8 June 2015 (53 spec., 8 dissected) by T. Peretolchina, and July 2017 (5 specimens, 1 dissected) by T. Sitnikova.

The 60 adult specimens of the new species were compared with seven individuals of the *Gyraulus acronicus* (A. Férussac, 1807) collected in a small inflow of the Krestovka River (51°51'44"N, 104°51'11"E) on 13 October 2015 and 2 October 2017 by T. Sitnikova; 4 of these specimens were dissected. The holotype and paratypes of the newly described species were deposited in the collection of the Zoological Institute RAS (St. Petersburg), registration numbers are 522-2015 (1) for the holotype and 522-2015 (2) for three paratypes . An additional 28 paratypes were deposited in the gastropod collection of the Limnological Institute SB RAS (Irkutsk, Russia) under Nos: 901, 902, 1101, and 1102.

Anatomical study and molecular analyses were performed on snails fixed in 80% ethanol that was changed for 70% ethanol after one day. Eight snails were photographed and the shells of 12 individuals were dissected. DNA was extracted from the feet; the teeth of radula were SEM-photographed and the soft tissues were dissected under a light stereomicroscope. Morphological study and descriptive terminology are based on the review of morphological characters of planorbid gastropods (Meier-Brook 1964, 1983; Brown 2001; Glöer 2002). Measurements of the shells were performed using the Image-Pro-Plus program for Windows XP.

Genomic DNA was extracted from muscle tissue using a modified method from Sokolov (2000). Gene fragments of mitochondrial cytochrome c oxidase subunit 1 (CO1) were amplified using primers L1490 (5’ – GGTCAACAAATCATAAAGATATTGG – 3’) and H2198 (TAAACTTCAGGGTGACCAAAAAATCA - 3’) (Folmer et al., 1994) and mitochondrial large ribosome subunit (16S) were amplified using primers ARL (5’ – CGCCTGTTTATCAAAAACAT – 3’) and BRH (5’ – CCGGTCTGAACTCAGATCACGT – 3’) (Palumbi et al. 1996). An average of 1-3 μL of extracted DNA was amplified in a 25 μL of PCR-mix using BioMaster HS-*Taq* PCR Kit (Biolabmix, Russia) following the manufacturer’s recommendations. Conditions of 30 cycles of amplification for both gene fragments were pre-denaturation at 94 °C for 5 min, followed by denaturation at 94 °C for 40 s, annealing of primers at 50 °C for 40 s, elongation at 72 °C for 60 s, and a final elongation step at 72 °C for 8 min. The reaction products were analyzed in 1% agarose gel. After electrophoresis, visible bands of the expected size were excised and then amplicons were cleaned up according to Maniatis et al. (1982). Sequencing was carried out in an ABI 3130 automated sequencer. The DNA sequences obtained were aligned using default settings by ClustalW (Thompson et al. 2002) and edited using the BioEdit software package (Hall 1999). All sequences were deposited in GenBank under accession numbers (Table 1). Additional sequences of other representatives of *Gyraulus* retrieved from GenBank are also listed in Table 1.

**Table 1.** Taxa used for phylogenetic analyses, with their GenBank Accession Numbers and references.

Species name	COI GB#	16S GB#	References
*G. albus*	KC495835	KC495952	Oheimb et al. (2013)
*G. crista*	KC495836	KC495953	
*G. rossmaessleri*	KC495714	KC495844	
*G. connollyi*	KC495776		
*Gyraulus* sp.	KC495834	KC495951	
*G. convexiusculus*	KF966542		Unpublished
*G. acronicus*, Krestovaya River			Present study
a435	MG773536		
a430	MG773535	MG800654	
*Gyraulus takhteevi* sp. n. (haplotype 1)			Present study
401_n	MG773534		
a437_w	MG773537		
442_w	MG773541		
443_w	MG773542		
439_n	MG773539		
438_n	MG773538		
444_w	MG773543		
441_n	MG773540		
445_w	MG773544		
*Gyraulus takhteevi* sp. n. (haplotype 2)			Present study
425 _w	MG773531		
429_w	MG773533		
427_w	MG773532	MG800655	

Mean pairwise, inter-specific *p*-distances between COI and 16S sequences were calculated using MEGA 6 (Tamura et al. 2013) (Table 3).

Phylogenetic reconstructions for COI mtDNA was inferred following a Bayesian method of phylogenetic inference as implemented by MrBayes v. 3.2.2 (Ronquist and Huelsenbeck 2003). Posterior probabilities of phylogenetic trees were estimated by a 15,000,000 generation Metropolis-coupled Markov chain Monte Carlo simulation (two runs with four chains) under the GTR+I+G model of substitution, determined as a best fit model using jModelTest v.2.1 (Darriba et al. 2012). A 50 % majority-rule consensus tree was constructed following a 25% burn-in of all sampled trees to allow likelihood values to reach stationary equilibrium.

## Taxonomy

### Family Planorbidae Rafinesque, 1815

#### Genus *Gyraulus* Charpentier, 1837

##### Gyraulus takhteevi sp. n.

Taxon classificationAnimaliaHygrophilaPlanorbidae

http://zoobank.org/975FA23C-CF59-44E2-91CC-A68285601B06

Gyraulus cf. borealis : Sitnikova and Takhteev 2006: 143 (record from thermal spring Khakusy)

###### Type locality.

Thermal spring Khakusy (East Siberia).

###### Types.

Holotype (dry) registration number in ZIN collection (St. Petersburg, Russia) is 522-2015 (1), 3 paratypes (dry) registration number is 522-2015 (2) with a label: ‘East-northern shore of Lake Baikal, thermal spring Khakusy, pebbles, water temperature 23–25 °C, #0957, col. T. Sitnikova, 09.08.2009’. Collections of the Limnological Institute SB RAS (Irkutsk, Russia): 2 paratypes (dry) under numbers 901 and 902 with the label: ‘Khakusy, shallow springs at a depth down to 3 cm, water temperature 33 °C, col. V. Takhteev, #57, 07.10.2004’; 20 paratypes (in alcohol) under number 1101 with a label ‘Khakusy, #1526, 08.06.2015, col. T. Peretolchina’ and 4 paratypes (dry) and 2 paratypes (in alcohol) under number 1102 collected 3 July 2003, #0344, T. Sitnikova.

###### Etymology.

The species name ‘*takhteevi*’ is in honor of the Russian zoologist and hydrobiologist Prof. V.V. Takhteev (Irkutsk State University) who investigates biota of thermal springs in East Siberia.

###### Description.

**Figure 1.**
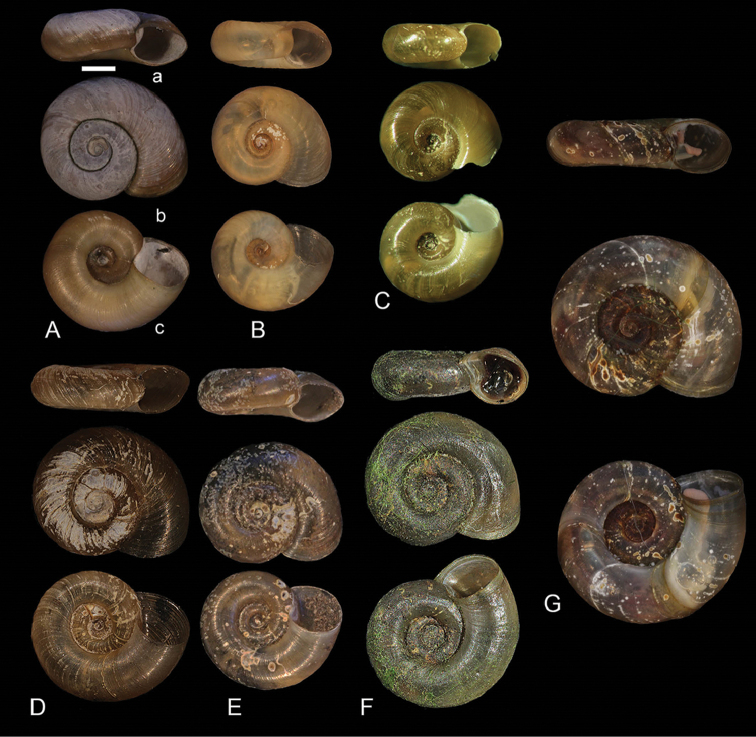
Shells of type specimens of *Gyraulus takhteevi* sp. n. and *G. acronicus* Férussac, 1807). **A** Holotype *G. takhteevi* sp. n. **B–E** Paratypes *G. takhteevi* sp .n. **A–C** morph 1 with narrow spire **D, E** morph 2 with wide spire **F–G** *G. acronicus* from Krestovka River: **F** young individual **F** mature individual after 5 months of a cultivation. Abbreviations: a – aperture view; b – right side with umbilicus, c – left side with spire. Scale bar 1 mm.

*Shell* (Fig. 1A–E) brown or green-brown, discoidal, pseudodextral, small, up to 5.0 mm of diameter at 4.0 whorls, smooth with fine growth lines on rounded last whorl, spire convex with rounded whorls, two last whorls of an umbilicus almost flat. Index a/b 0.36 – 0.57. Height of last whorl (a) less than aperture width. Aperture oval rounded. Species occurs in two morphs differing in spire width, narrow (Fig. 1A–C) and wide (Fig. 1D–E); all small individuals (less than three whorls) have a narrow spire; at 3.25 – 4.0 whorls the portion of morph 2 (with wide spire) is approximately 1/3 or 1/2 of total number of examined adult specimens. The designated holotype belongs to the dominant morph 1 (with a narrow spire). Sizes of the holotype and paratypes are presented in Table 2.

**Table 2.** Shell dimensions and whorl counts of type specimens of *Gyraulus takhteevi* sp. n. Abbreviations: SH – height of shell; SW– width of shell; b – width of last whorl without aperture; a – height of this whorl; SpW – width of spire; AW – width of aperture; AH – height of aperture; n – number of whorls. Measurements are in mm except for number of whorls.

Specimens/Character	SW	SH	SpW	b	a	AW	AH	n
Holotype	4.31	1.68	1.23	3.01	1.27	1.67	1.20	3.75
Paratypes (narrow spire)	4.03	1.42	1.25	2.78	1.21	1.64	1.25	3.75
	3.57	1.5	1.10	2.4	1.17	1.67	1.42	3.5
	3.45	1.5	1.0	2.37	1.12	1.26	1.19	3.25
(wide spire)	5.2	1.6	1.93	3.8	1.3	1.5	1.45	4.0
	4.45	1.7	1.54	3.24	1.18	1.5	1.31	3.75
	4.1	1.65	1.3	2.9	1.1	1.5	1.4	3.5

**Table 3.** Pairwise p-distances (%) between COI sequences of different species of the genus *Gyraulus*.

Taxa	1	2	3	4	5	6	7	8	9
**1.** *G. acronicus* Krestovaya									
**2.** *Gyraulus takhteevi* sp. n.(haplotype 1)	0.8								
**3.** *G. takhteevi* sp. n. (haplotype 2)	1.0	0.2							
**4.** *Gyraulus* sp. KC495834 (Lake Baikal)	0.7	1.5	1.3						
**5.** *G. albus*	6.9	7.0	6.9	6.9					
**6.** *G. rossmaessleri*	11.0	11.0	10.8	10.6	11.3				
**7.** *G. convexius*	10.5	10.8	10.8	10.6	7.4	12.6			
**8.** *G. crista*	12.3	12.6	12.6	12.4	12.8	11.6	12.4		
**9.** *G. connollyi*	11.5	11.5	11.3	11.5	8.8	12.6	9.0	14.7	

**Figure 2.**
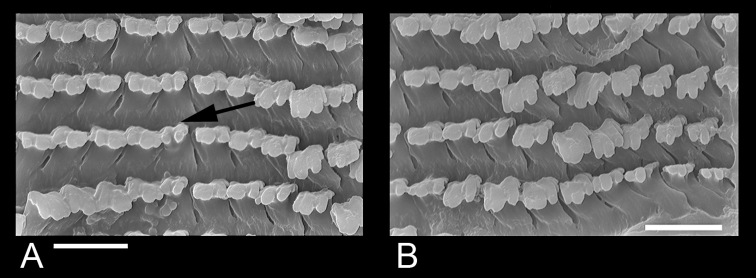
Radular teeth of *Gyraulus takhteevi* sp. n. **A** Central and lateral teeth (arrow shows a central tooth) **B** Marginal teeth. Scale bar 10 mμ.

*Radular teeth*. The formula of the radula is 10 (9)–1–(9) 10. The central radular teeth are bicuspid with two equal-sized cusps. Two first lateral teeth usually bicuspid, sometimes with third small cusp. Other teeth have three rounded cusps, and only young (not working or worn) tricuspid teeth have three long sharp cusps. Mesocone is flanked (Fig. 2). Both morphs have identical radular morphologies and formulae.

**Figure 3.**
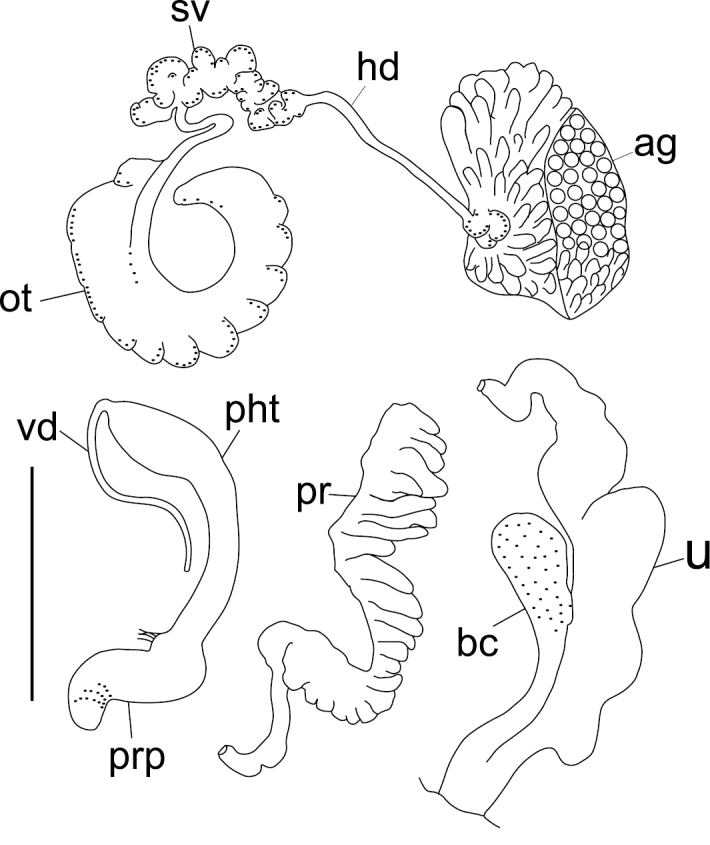
Drawing pictures of the reproductive organs of *Gyraulus takhteevi* sp. n. Abbreviations: **ag** albumen gland **bc** bursa copulatrix **hd** hermaphrodite duct **ot** ovotestis **pht** phallotheca **pr** prostate **prp** preputium **sv** seminal vesicles **vd** vas deferens **u** uterus. Scale bar 1 mm.

**Figure 4.**
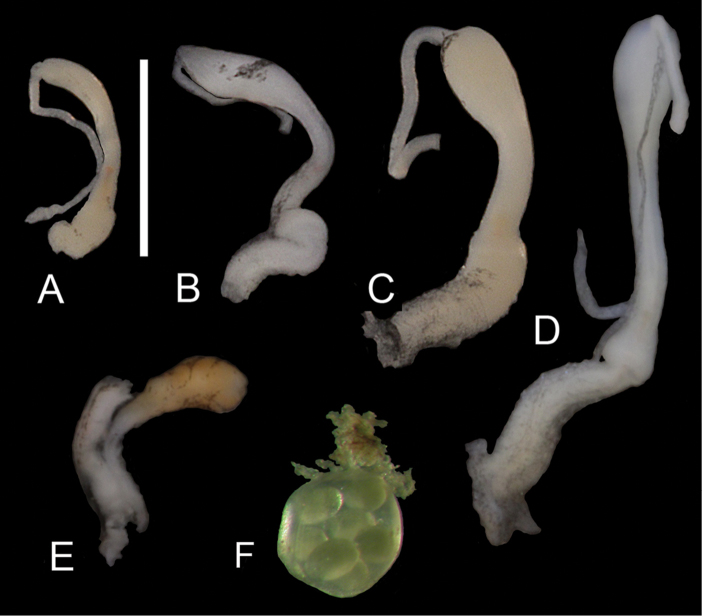
Copulatory organs (**A–D**), bursa copulatrix (**E**) and egg mass (**F**). **A–B, E–F** *Gyraulus takhteevi* sp. n.: **A** morph 1 **B** morph 2 **C–D** *G. acronicus* from Krestovka River: young (shell Fig. 1F), male stage of mature (shell Fig. 1G).

**Figure 5.**
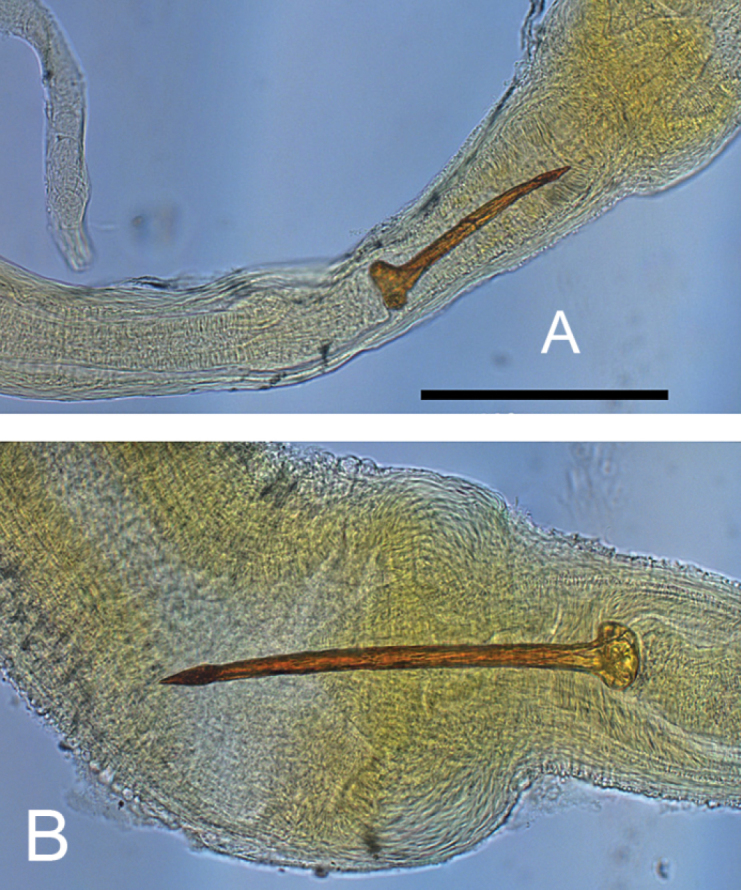
Stylets of *Gyraulus takhteevi* sp. n. (**A**) and *G. acronicus* from Krestovka River (**B**). Scale bar 200 mμ.

*Reproductive system*. Seminal vesicles have thickened bend before joining with hermaphrodite duct, which is thin up to carrefour. Prostate consists of 22–26 diverticula, bursa copulatrix is oval in shape, its length including duct more than ½ length of oviduct. Length of copulatory organ almost equal to length of prostate (Fig. 3). Phallotheca twice as long as preputium length in morph with narrow spire and 1.7–1.8 times in morph with wide spire (Fig. 4A, B). Preputium slightly turned-up. Seminal pore lies near the proximal end of the thickening. Length of the stylet (Fig. 5A) varies from 189 to 260 mµ (n = 5).

Egg mass (= cluster or syncapsula) is a round transparent sac less than 1 mm in size, consisting of 4-5 eggs (Fig. 4F).

###### Differential diagnosis.

The new species (especially morph 2 with wide spire) is similar to the Palaearctic species *G. acronicus* (Fig. 1F, G) in shell shape, and differs from it in the small size of adults, which do not reach 6–7 mm diameter. Additionally, the new species differs from *G. acronicus* in having shorter preputium, the elongated oval shape of the bursa copulatrix, smaller number of prostate folds, and the smallest stylet length. A preputium of *G. acronicus* is 1.3–1.7 times shorter than the phallotheca (Fig. 4C, D); the bursa copulatrix has an elongate club-shaped (Meier-Brook, 1964, 1983) or a wide rounded shape, and length of copulatory organ is less than prostate length (Glöer and Vinarski 2009; own data). The length of stylet of *G. acronicus* is more than 300 mµ (Fig. 5B). The size of the egg mass of *G. takhteevi* sp. n. is less than that of *G. acronicus* (its length up to 2.05 mm) and consists of fewer eggs (4–5 eggs vs. 5–7 eggs in *G. acronicus*) (Beriozkina and Starobogatov 1988).

###### Distribution and ecology.

Snails similar to morph 1 of the new species were also found in the thermal spring Frolikha located near to Khakusy (ca. 20 km north); all of them were young and were not examined in detail. The number of gastropods in the thermal spring Khakusy in March 2016 was 1,706 individuals/m^2^ at water temperature +29 °C in a locality 2 m far from the main source; a minimal number of snails (59 indv./m^2^) was registered at water temperature +10 °C in a small pond downstream of the main source (Epova et al. 2017). The proportions of *G. takhteevi* sp. n. and *Lymnaea* were about fifty-fifty.

The confinement of morphs to different sites of the thermal spring has not been confirmed. The population of *G. takhteevi* consists mainly of young snails, in which the morph with a narrow spire dominated: in July 2003 28 of the 39 collected snails were juvenile, with adults presented by six snails with shells of narrow spire (morph 1) and five individuals with wide spire (morph 2). In June 2015 there were 16 individuals of morph 1 and eleven specimens of morph 2, with more than 50 young snails; in March 2016 39 young specimens were collected and 16 adult snails of morph 1 and six individuals of morph 2.

The cultivation of the new species under summer conditions of ephemeral waterbodies (sand, pebbles, water temperature +20–24 °C, food items of vegetable fodder) was not successful, and all snails died in two weeks. It is worth noting that specimens of *G. acronicus* under the same conditions lived more than one year and reproduced, attached their egg masses to pebbles. According to the interruption lines on shells, the snails live up to 5 years.

###### Sequences analysis.

**Figure 6.**
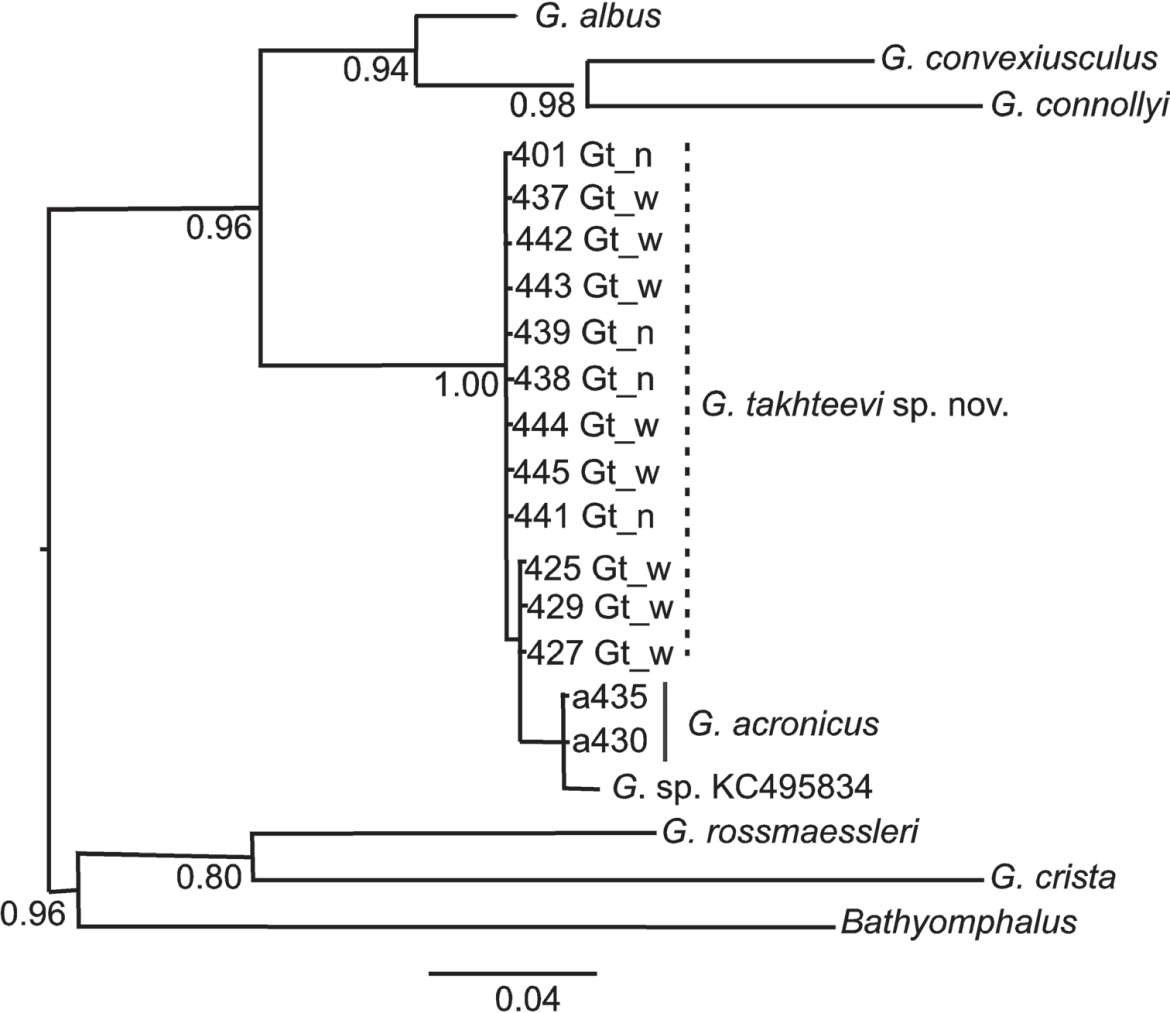
Phylogram derived from Bayesian analysis of CO1 mtDNA gene sequences. Values at nodes represent posterior probabilities. The scale bar represents the branch length as a measure of substitution per site.

A total 14 COI (620 bp long) and 14 16S (500 bp long) sequences were produced. Inspection of the sequences revealed the existence of two unique haplotypes for COI among *G. takhteevi* sp. n. These haplotypes weakly differ from each other (in 1–2 nucleotide substitutions). Both morphs (shells with narrow or wide spire) are part of the haplotype 1, while haplotype 2 consists of the morph with wide spire shell (Fig. 6). There are no genetic differences between 16S nucleotide sequences obtained.

The haplotype of *G. acronicus* from Krestovka River shares the same clade as *Gyraulus* sp., sequence of which is retrieved from GenBank (KC495834), which differs from that of *G. takhteevi* sp. n. with 5–6 nucleotide substitutions (~ 1%).

## Discussion

Despite the small genetic distances between *G. takhteevi* sp. n. and *G. acronicus*, morphological differences between them (shell size of mature individuals, maximal number of prostate folds, length of the phalloteka relative to length of the prepuceum, length of a stylet, shape of the bursa copulatrix) allow one to consider them as separate species. Moreover, the new species demonstrates significant adaptation to a specific habitat of the thermal spring Khakusy. The water in the spring does not freeze in winter, the temperature of the water changes very little, and the food items like microorganisms, bacterial mats, and vegetable detritus are present during all seasons of a year. However, the two co-existent morphs of *G. takhteevi* sp. n. are likely to correspond to two interbreeding generations, hatching from eggs, growth and reproduction of which are confined to different seasons. The co-existence of both morphs appears to be constantly maintained, since they have been found over several years of the investigation.

The low level of genetic distances between *G. takhteevi* sp. n. and *G. acronicus* from the Krestovka River indicates that a recent divergence of the species happened after the glaciers covering large areas of the northeast coast of Baikal during last glacial period started to melt about 18 kyr BP (Osipov and Khlystov 2010). The adaptation to the thermal conditions of ‘a closed habitat’ formed by island isolation under selective pressures led to an interruption of gene exchange between *G. takhteevi* sp. n. and *G. acronicus*, resulting in differences in their morphological and physiological characteristics, and ecological preferences.

## Supplementary Material

XML Treatment for Gyraulus takhteevi
